# Opening the black box of artificial intelligence for clinical decision support: A study predicting stroke outcome

**DOI:** 10.1371/journal.pone.0231166

**Published:** 2020-04-06

**Authors:** Esra Zihni, Vince Istvan Madai, Michelle Livne, Ivana Galinovic, Ahmed A. Khalil, Jochen B. Fiebach, Dietmar Frey

**Affiliations:** 1 Charité Lab for Artificial Intelligence in Medicine—CLAIM, Charité - Universitätsmedizin Berlin, Berlin, Germany; 2 Centre for Stroke Research Berlin, Charité - Universitätsmedizin Berlin, Berlin, Germany; University of Craiova, ROMANIA

## Abstract

State-of-the-art machine learning (ML) artificial intelligence methods are increasingly leveraged in clinical predictive modeling to provide clinical decision support systems to physicians. Modern ML approaches such as artificial neural networks (ANNs) and tree boosting often perform better than more traditional methods like logistic regression. On the other hand, these modern methods yield a limited understanding of the resulting predictions. However, in the medical domain, understanding of applied models is essential, in particular, when informing clinical decision support. Thus, in recent years, interpretability methods for modern ML methods have emerged to potentially allow explainable predictions paired with high performance. To our knowledge, we present in this work the first explainability comparison of two modern ML methods, tree boosting and multilayer perceptrons (MLPs), to traditional logistic regression methods using a stroke outcome prediction paradigm. Here, we used clinical features to predict a dichotomized 90 days post-stroke modified Rankin Scale (mRS) score. For interpretability, we evaluated clinical features’ importance with regard to predictions using deep Taylor decomposition for MLP, Shapley values for tree boosting and model coefficients for logistic regression. With regard to performance as measured by Area under the Curve (AUC) values on the test dataset, all models performed comparably: Logistic regression AUCs were 0.83, 0.83, 0.81 for three different regularization schemes; tree boosting AUC was 0.81; MLP AUC was 0.83. Importantly, the interpretability analysis demonstrated consistent results across models by rating age and stroke severity consecutively amongst the most important predictive features. For less important features, some differences were observed between the methods. Our analysis suggests that modern machine learning methods can provide explainability which is compatible with domain knowledge interpretation and traditional method rankings. Future work should focus on replication of these findings in other datasets and further testing of different explainability methods.

## Introduction

Machine learning (ML) techniques are state-of-the-art in predictive modeling in fields like computer vision and autonomous navigation [1]. Increasingly, these tools are leveraged for clinical predictive modeling and clinical decision support, where clinical values are used to predict a clinical status, e.g. a diagnosis, outcome or risk [2,3]. Here, newer machine learning techniques—we will refer to them as modern machine learning techniques in this work—including artificial neural nets (ANN), especially deep learning (DL), and ensemble models such as tree boosting have often shown higher performance than traditional machine learning techniques such as linear or logistic regression, e.g. [4–8].

However, a common criticism of these modern techniques is that while they might increase model performance they do not provide the possibility to explain the resulting predictions [9]. In contrast, traditional techniques allow explanations by various means and this approach has been the backbone of explainable clinical predictive modeling to date [10]. The necessity of interpretable ML systems are of particular concern in the medical domain. An explainable AI system is essential to provide: 1) Interpretation and safe check of the acquired results during development [11]. 2) Better assessment of safety and fairness of medical products, especially regarding bias, during the regulatory process [12]. 3) Domain knowledge supported interpretation leading to increased trust by the physicians, other healthcare professionals, and patients [12]: Some argue that black box approaches are unacceptable for clinical decision support from the physician´s point-of-view [13] and from the patient's point-of-view [14]. Thus, currently, researchers and developers are facing an unfortunate trade-off: either to use methods with potentially higher performance or to use methods providing explainability to comply with ethical and regulatory requirements [9].

Fortunately, interpretability methods tailored to modern machine learning algorithms have emerged lately, therefore potentially allowing high performance and explainable models. For one, in the last few years several techniques have been developed to open the most notorious black box, namely artificial neural networks and provide explainable models [11]. Moreover, tree boosting provides high performance clinical predictive modeling and also allows the calculation of feature importance and ranking, e.g. Lundberg et al [15]. However, to our knowledge, these approaches have not yet been compared to the traditional methods in terms of interpretability for clinical predictive modeling.

In the present work, we thus compared the above mentioned two modern ML methods, ANNs and tree boosting, to traditional methods with regard to explainability. We chose a well-characterized stroke clinical outcome paradigm. Here, available clinical features such as age, the severity of the stroke or information about treatment are used to predict the 3 months post-stroke outcome. Many replications in the past have established main factors driving the prediction, namely age and stroke severity, e.g. [16–19]. Thus, within this paradigm, modern machine learning explanations can be interpreted against a baseline. Concretely, we used a multilayer perceptron (MLP) with deep Taylor decomposition as an example for an explainable ANN approach [20], the CATBOOST algorithm with Shapley Additive exPlanations (SHAP) values as an example for explainable tree boosting [15] and compared performance and explainability with different versions of (regularized) logistic regression for a binary outcome (GLM, LASSO, and Elastic Net).

## Methods

### Patients and clinical metadata pre-processing

In a retrospective analysis, patients with acute ischemic stroke from the 1000plus study were included [21]. The study was approved by the institutional ethics committee of Charité Universitätsmedizin Berlin in accordance with the Helsinki declaration and all patients gave written informed consent. Patients were triaged into receiving iv-tissue-plasminogen-activator (tPA) for thrombolysis therapy or conservative therapy. The modified Rankin Scale (mRS), representing the degree of disability or dependence in the daily activities, was assessed for each patient 3 months post-stroke via a telephone call. The available database consisted of 514 patients who received imaging at 3 imaging time points. Of these, 104 were lost-to-follow-up and had no mRS values. 1 patient had to be excluded due to values outside of the possible parameter range. Moreover, 95 patients had to be excluded due to infratentorial stroke and no visible diffusion-weighted imaging (DWI) lesions. Specific further inclusion criteria of our sub-study were a ratio of at least 1 to 4 for binary variables (absence/presence) and no more than 5% missing values resulting in the final number of 314 patients and the following clinical parameters for the predictive models: age, sex, initial NIHSS (National Institute of Health Stroke Scale; measuring stroke severity), history of cardiac disease, history of diabetes mellitus, presence of hypercholesterolemia, and thrombolysis treatment. For a summary of the patients' clinical features and their distribution, see Table 1.

**Table 1:** Summary of the clinical data.

Clinical information	Value
Median age (IQR)	72 (15)
Sex (Females/ Males)	196 / 118
Median initial NIHSS (IQR)	3 (5)
Cardiac history (yes/ no)	84 / 230
Diabetes mellitus (yes/ no)	79 / 235
Hypercholesterolemia (yes/ no)	182 / 132
Thrombolysis (yes / no)	74 / 240

The table summarizes the distribution of the selected clinical data covariates acquired in the acute clinical setting. NIHSS stands for National Institutes of Health Stroke Scale; IQR indicates the interquartile range.

### Data accessibility

Data cannot be shared publicly because of data protection laws imposed by institutional ethics committee guidelines. Data might be available from the institutional ethics committee of Charité Universitätsmedizin Berlin (contact via ethikkommission@charite.de) for researchers who meet the criteria for access to confidential data. The code used in the manuscript is available on Github (https://github.com/prediction2020/explainable-predictive-models).

### Outcome prediction supervised machine learning framework

In a supervised machine-learning framework, the clinical parameters (Table 1) were used to predict the final outcome of stroke patients in terms of dichotomized 3-months post-stroke mRS, where mRS ϵ {0,1,2} indicates a good outcome (i.e. class label for a given observation *i*) and mRS ϵ {3,4,5,6} indicates a bad outcome (i.e. class label for a given observation *i*). The applied dichotomization resulted in 88 positive (i.e. bad outcome) and 226 negative (i.e. good outcome) classes.

### Feature multicollinearity

Importantly, methods for feature ranking can be influenced by feature multicollinearity. Particularly, Beta weights in regression analysis can be erroneous in case of multicollinearity [22,23] and certain applications of feature importance calculation for tree boosting are simplified under the assumption of feature independence. To ensure an unbiased comparison of the models interpretability we estimated multicollinearity of the features using the variance inflation factor (VIF) [24]. The chosen features in the analysis demonstrated negligible multicollinearity with VIFs < 1.91 (Age: 1.15; Sex: 1.91, NIHSS: 1.28; Cardiac history: 1.33; Diabetes: 1.36; Hypercholesterolemia: 1.74; Thrombolysis: 1.50). This makes our stroke outcome paradigm particularly suited to compare explainability.

### Predictive modeling and Interpretability

In this study, machine-learning (ML) methods were applied to predict the final outcome based on clinical data. In the context of tabular data as in the given study, the interpretability of the resulting models corresponds to a rating of feature importance. The interpretability frameworks suggested in this study are tailored to the models and therefore indicate the relative contribution of the features to the respective model prediction. The different ML algorithms and the corresponding interpretability derivations are described as follows.

#### Traditional (linear) ML frameworks

*1*. *Generalized Linear Model (GLM)*. GLM is a generalization of linear regression that allows for a response to be dichotomous instead of continuous. Hence the model predicts the probability of a bad outcome (vs. good outcome) based on a set of explanatory variables according to the following relation:

$$P\left(O=1|\bar{X}\right)=\frac{1}{1+e^{-\sum_{i}\beta_{i}x_{i}}}$$

where $P\left(O=1|\bar{X}\right)$ is the probability for a bad outcome (*O* = 1) given the vector of corresponding covariates $\bar{X}$.

*β* stands for model parameterization. The objective function for the optimization problem is defined by maximum likelihood estimation (MLE):

$$J\left(\bar{\beta }\right)=ln\prod_{i=1}^{N}P(O_{i}=1|{\bar{X}}_{i},\bar{\beta })$$

where $J\left(\bar{\beta }\right)$ stands for the objective function for the given model parametrization, $P(O_{i}=1|{\bar{X}}_{i},\bar{\beta })$ is the predicted outcome probability for the given covariates ${\bar{X}}_{i}$ and model parametrization *β* and *N* is the number of observations. In this formulation, this special case of a GLM is also known as logistic regression.

*2*. *Lasso*. Lasso, standing for least absolute shrinkage and selection operator, provides the L1 regularized version of GLM. An L1 penalization of the model parametrization reduces overfitting of the model and is applied by the addition of the L1 regularization term to the objective function:

$$J_{L}\left(\bar{\beta }\right)=J\left(\bar{\beta }\right)+\alpha \|\bar{\beta }\|$$

where $J_{L}\left(\bar{\beta }\right)$ stands for the Lasso objective function and *α* is the scaling factor hyperparameter.

*3*. *Elastic Nets*. Similarly to Lasso, elastic net provide a regularized variate of the GLM. Here two types of regularization terms are added to the objective function that provide L1 and L2 penalization of the model parametrization respectively:

$$J_{EN}\left(\bar{\beta }\right)=J\left(\bar{\beta }\right)+\alpha \|\bar{\beta }\|+\gamma \|\bar{\beta^{2}}\|$$

where $J_{EN}\left(\bar{\beta }\right)$ stands for the elastic nets objective function and *α* and *γ* are the scaling factors hyperparameters.

For the three linear models, the interpretability of the models was deduced using the resulted model parametrization. Hence, the rating of the features was derived by the values of the model coefficients *β*. As outlined above, this is sufficient since our features do not exhibit collinearity [23].

#### Modern (nonlinear) ML frameworks

*4*. *Tree boosting (CatBoost)*. Tree boosting solves the described classification problem by producing a prediction model as an ensemble of weak classification models, i.e. classifiers. As an ensemble method, the algorithm builds many weak classifiers in the form of decision trees and then integrates them into one cumulative prediction model to obtain better performance than any of the constituent classifiers. The prediction is then given using K additive functions:

$$P\left(O=1|\bar{X}\right)=\sum_{k=1}^{K}f_{k}\left(\bar{X}\right),f_{k}\in F$$

where $F=\left\{f\left(x\right)=w_{q\left(x\right)}\right\}\left(q:{\mathbb{R}}^{m}\overset{□}{\to }T,\text{w}\epsilon {\mathbb{R}}^{T}\right)$ is the space of regression trees. Here *q* denotes the structure of each tree and *T* is the number of leaves in the tree. Each f(x) represents an independent tree structure *q* and leaf weights *w*. The output of the regression trees is a continuous score represented by *w*_*i*_ for leaf *i*. Each observation is classified using each constituent tree to the corresponding leafs and the outcome prediction $P\left(O=1|\bar{X}\right)$ is finally calculated as the cumulative sum of scores of the corresponding leafs. The objective function for optimization constitutes of the convex loss function, here chosen as logistic function, and a regularization component:

$$J_{c}\left(\varphi \right)=\sum_{i}l\left(y_{i}^{'},y_{i}\right)+\sum_{k}\Omega \left(f_{k}\right)$$

where the convex loss is given by:

$$l\left(y_{i}^{'}=P\left(O=1|\bar{X}\right),y_{i}\right)=\frac{-\sum_{i=1}^{N}w_{i}\left(y_{i}\log\left(y_{i}^{'}\right)+\left(1-y_{i}\right)\log\left(1-y_{i}^{'}\right)\right)}{\sum_{i=1}^{N}w_{i}}$$

which is the logistic loss and the regularization component is given by:

$$\Omega \left(f\right)=\gamma T+\frac{1}{2}\lambda {\left\|w\right\|}^{2}$$

where *ω* are the model weights penalized through L2 normalization and *T* is again the number of leaves in the tree. Here *φ* represents the corresponding model parametrization. In this study we used the CATBOOST module to implement the tree boosting model allowing to successfully integrate both numerical and categorical features [25].

In the context of tree boosting models, SHapley Additive exPlanations (SHAP) values construct a robust unified interpretability framework, breaking down the prediction to show the impact of each input feature [15]. The SHAP values attribute to each feature the average change in the model prediction when that feature is integrated to the model. It calculates a marginal contribution of the feature by averaging over every possible sequence in which that feature could have been introduced to make the prediction. This allows for calculating the contribution of the feature to the final decision irrespective of in which order it was used in the decision tree. The Shapley value of an input feature i for a single observation is calculated as follows:

$$\phi_{i}=\sum_{S\subseteq F\left\{i\right\}}\frac{\left|S\right|!\left(\left|F\right|-\left|S\right|-1\right)!}{\left|F\right|!}\left[f_{S\cup^{\;}\left\{i\right\}}\left(x_{S\cup^{\;}\left\{i\right\}}\right)-f_{S}\left(x_{S}\right)\right]$$

where F is the set of all input features, |F| representing its size. S represents any subset of input features that was introduced to the model before feature i, and |S| is the size of that subset. The second factorial in the nominator then gives the size of the remaining subset of input features that will succeed feature i. The final multiplicative factor quantifies the difference in the model prediction when feature i is introduced.

Finally, the overall rating of the feature contribution to the model is then achieved by averaging the SHAP values over all observations.

*5*. *MLP*. A multilayer perceptron (MLP) is a type of feedforward artificial neural network that is composed of connectionist neurons, also known as perceptrons, in a layered structure. An MLP architecture is constructed of 3 components: 1) an input layer to receive the information 2) an output layer that makes a decision or prediction about the input and 3) one or more hidden layers that allow for feature extraction and modeling of the covariates dynamics using nonlinear transformations. According to the universal approximation theorem, an MLP with one hidden layer can approximate any function [26].

Here the model prediction is given by:

$$P\left(O=1|\bar{X}\right)=f\left(g\left(a\left(g\left(\bar{X}\right)\right)\right)\right)$$

where $f\left(x_{k}\right)=\frac{\text{exp}\left(x_{k}\right)}{\sum_{c}\text{exp}\left(x_{c}\right)}$ is the (softmax) output layer activation, *k* is the predicted class and *c* is any of the possible classes for prediction. denotes the hidden layer activation function where *M* represents the number of nodes in the layer.

The core objective function utilized for the MLP model was binary cross-entropy:

$$J_{m}\left(\varphi \right)=-\frac{1}{N}\sum_{i=1}^{N}y_{i}\log\left(y_{i}^{'}\right)+\left(1-y_{i}\right)\log\left(1-y_{i}^{'}\right)$$

where *φ* represents the corresponding model parametrization. Regularization of the model was entailed using: 1) L1 regularization, i.e. linear penalization of the model parametrization 2) dropout, i.e. random drop of nodes at each stage of the training process with a probabilistic rate *DR* and consecutive weighting of each of the nodes’ output with *(1-DR)* in the prediction inference to yield the expected value of the output.

Explainability techniques for ANNs can be grouped into two categories: gradient-based methods such as saliency [27] and backward propagation methods such as deconvolution [28], guided backpropagation [29], SmoothGrad [30] and layer-wise relevance propagation (LRP) [31]. Saliency is a simple technique that for a given data point identifies the most relevant input features to which the output is most sensitive. The advantage of saliency is the simplicity of the method application. However, it comes with the disadvantage of limited capability to provide explainability, due to its relation to local differential effects only. In comparison, backward propagation methods make use of the graph structure of neural networks by mapping the prediction backwards along each layer using a set of predefined rules and thus can provide better explanations to what made the network arrive at a particular decision [11]. Amongst these methods, LRP provides the advantage of introducing a conservation property during the propagation of relevance values and has shown an excellent benchmark performance [32]. For a specific set of rules, the LRP can be seen as computing a Taylor decomposition of the relevance at a layer onto its predecessor. This is called deep Taylor decomposition and has been proposed by Samek et al. as the method of choice for the backpropagation rule in LRP [11].

Deep Taylor decomposition is an interpretation of layer-wise relevance propagation when the parameters α and β in the propagation rule are set accordingly [20,31]. These parameters regulate the contribution of positive and negative connections between neurons to the relevance calculation. With α = 1 and β = 0, the relevance projected from a neuron k onto its input neuron j can be written by the following simpler rule which is equivalent to a first order Taylor decomposition:

$$r_{j\leftarrow k}=\frac{a_{j}w_{jk}^{+}}{\sum_{j}a_{j}w_{jk}^{+}}r_{k}$$

where *a*_*j*_ is the activation of neuron *j* and *w*_*jk*_^*+*^ is the positive weight between neurons *j* and *k*. Summing $r_{j\leftarrow k}$ over all neurons *k* to which neuron *j* contributes to yields the following propagation rule:

$$r_{j}=\sum_{k}\frac{a_{j}w_{jk}^{+}}{\sum_{j}a_{j}w_{jk}^{+}}r_{k}$$

All neuron relevance values are propagated layer-wise using this rule from the final output layer until the input, providing the input features with final relevance values.

The overall features importance was calculated as the weighted average of the observations with relation to the confidence of prediction:

$$R\left(f\right)=\frac{1}{N}\sum_{i=1}^{N}\theta_{i}r_{i}\left(f\right)$$

with $\theta_{i}=y_{i}\cdot P\left(O=1|{\bar{X}}_{i}\right)+\left(1-y_{i}\right)\left(1-P\left(O=1|{\bar{X}}_{i}\right)\right)$ where *R(f)* is the normalized feature rating and *r*_*i*_*(f)* is the feature contribution for the given MLP model for observation *i* using deep Taylor decomposition calculated by the propagation rule presented above.

### Models training and validation

The data were randomly split into training- and test sets with a corresponding 4:1 ratio. Mean/mode imputation and feature scaling using zero-mean unit variance normalization based on the training set was performed on both sets. To target class imbalance the training set was randomly sub-sampled to yield uniform class distribution. The models were then tuned using 10-folds cross-validation. The whole process was repeated 50 times (shuffles). Table 2 provides a summary of the tuned hyperparameters for each model.

**Table 2:** Summary of hyperparameters tuning.

Model	Hyperparameter	Range
LASSO	C (inverse of regularizer multiplier)	0.10, 0.12, 0.15, 0.18, 0.21, 0.26, 0.31, 0.37, 0.45, 0.54, 0.66, 0.79, 0.95, 1.15, 1.39, 1.68, 2.02, 2.44, 2.95, 3.56, 4.29, 5.18, 6.25, 7.54, 9.10,10.9, 13.3, 16.0, 19.3, 23.3, 28.1, 33.9, 40.9, 49.4, 59.6, 72.0, 86.9, 105, 126, 153, 184, 222, 268, 324, 391, 471, 569, 687, 829, 1000
Elastic net	L1 ratio	0, 0.05, 0.1, 0.15, 0.2, 0.25, 0.3, 0.35, 0.4, 0.45, 0.5, 0.55, 0.6, 0.65, 0.7, 0.75, 0.8, 0.85, 0.9, 0.95
	Alpha	0.00001, 0.00004, 0.00016, 0.0006, 0.0025, 0.01, 0.04, 0.16, 0.63, 2.5, 10
CatBoost	Tree depth	2, 4
	Learning rate	0.03, 0.1, 0.3
	Bagging temperature	0.6, 0.8, 1.
	L2 leaf regularization	3, 10, 100, 500
	Leaf estimation iterations	1, 2
MLP	Number of hidden neurons	5, 10, 15, 20
	Learning rate	0.001, 0.01
	Batch size	16, 32
	Dropout rate	0.1, 0.2
	L1 regularization ratio	0.0001, 0.001

The table details the hyperparameters and corresponding range that were tuned for each model in the cross-validation process.

### Performance assessment

The model performance was tested on the test set using receiver-operating-characteristic (ROC)-analysis by measuring the area-under-the-curve (AUC). The performance measure was taken as the median value over the number of shuffles.

### Interpretability assessment

The absolute values of the calculated feature importance scores were normalized, i.e. scaled to unit norm, in order to provide comparable feature rating across models: For each sample (each of the 50 shuffles) the calculated importance scores were rescaled to be confined within the range [0,1] with their sum equal to one. Then, for each feature the mean and standard deviation over the samples (shuffles) were calculated and reported as the final rating measures.

## Results

### Performance evaluation

**Fig 1:**
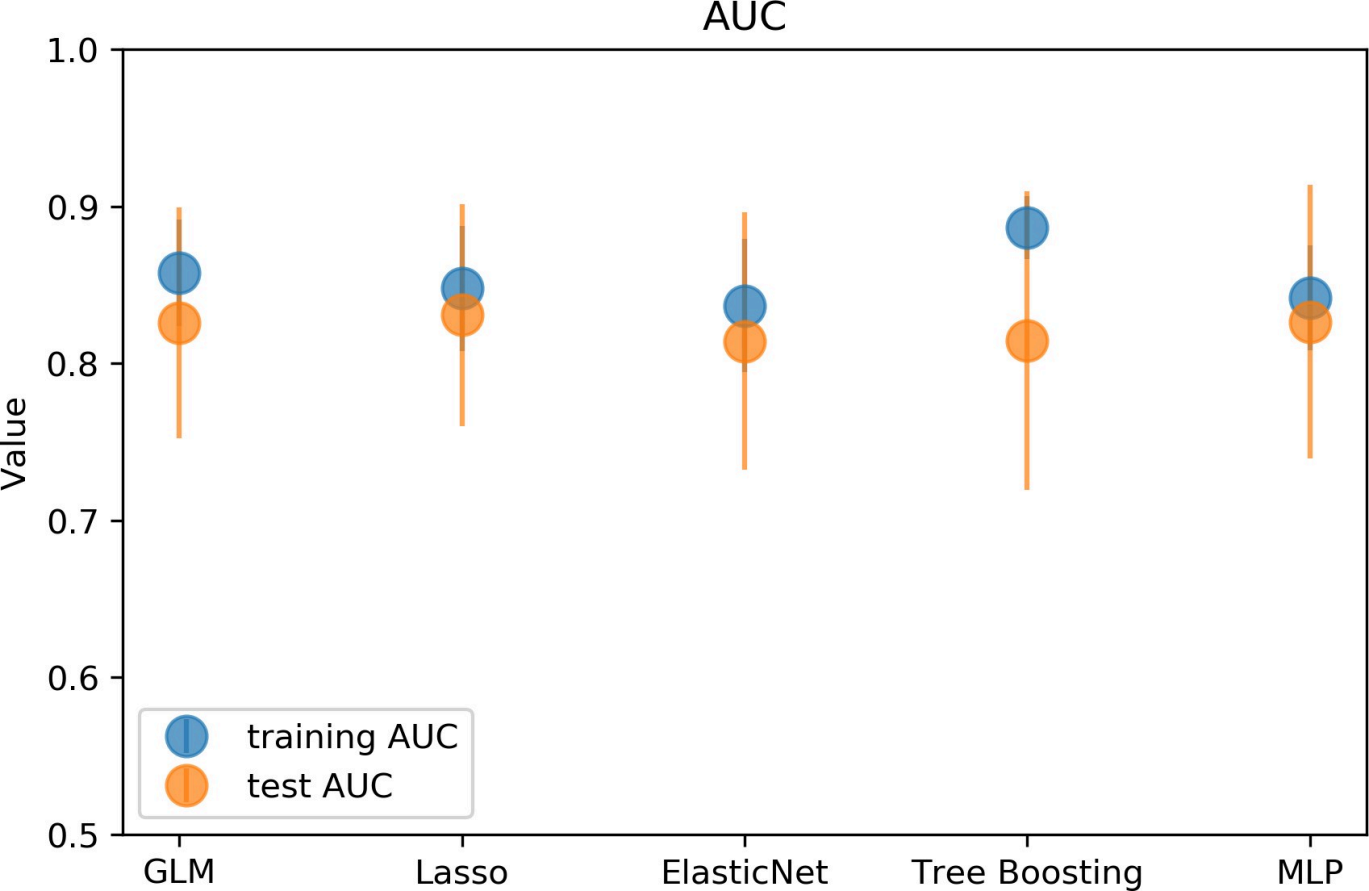
Graphical representation of the model performance results. The graph illustrates the performance of the different models evaluated on the training (blue) and test (orange) sets: generalized linear model (GLM), Lasso, Elastic net, Tree Boosting and multilayer perceptron (MLP). The markers show the median AUC over 50 shuffles and the error bars represent interquartile range (IQR). All models showed a similar median AUC around 0.82. The largest difference in performance between training and test set, indicating potential overfitting, was observed for the Catboost model.

All models demonstrated comparable performance for 3 months dichotomized mRS prediction as measured by AUC values on the test set: GLM 0.83, Lasso 0.83, Elastic Nets 0.81, Tree boosting 0.81 and MLP 0.83. While Catboost showed the highest performance, the difference to the other models was very small. For a graphical representation of the models performance on the training and test sets please see Fig 1.

### Interpretability analysis

**Fig 2:**
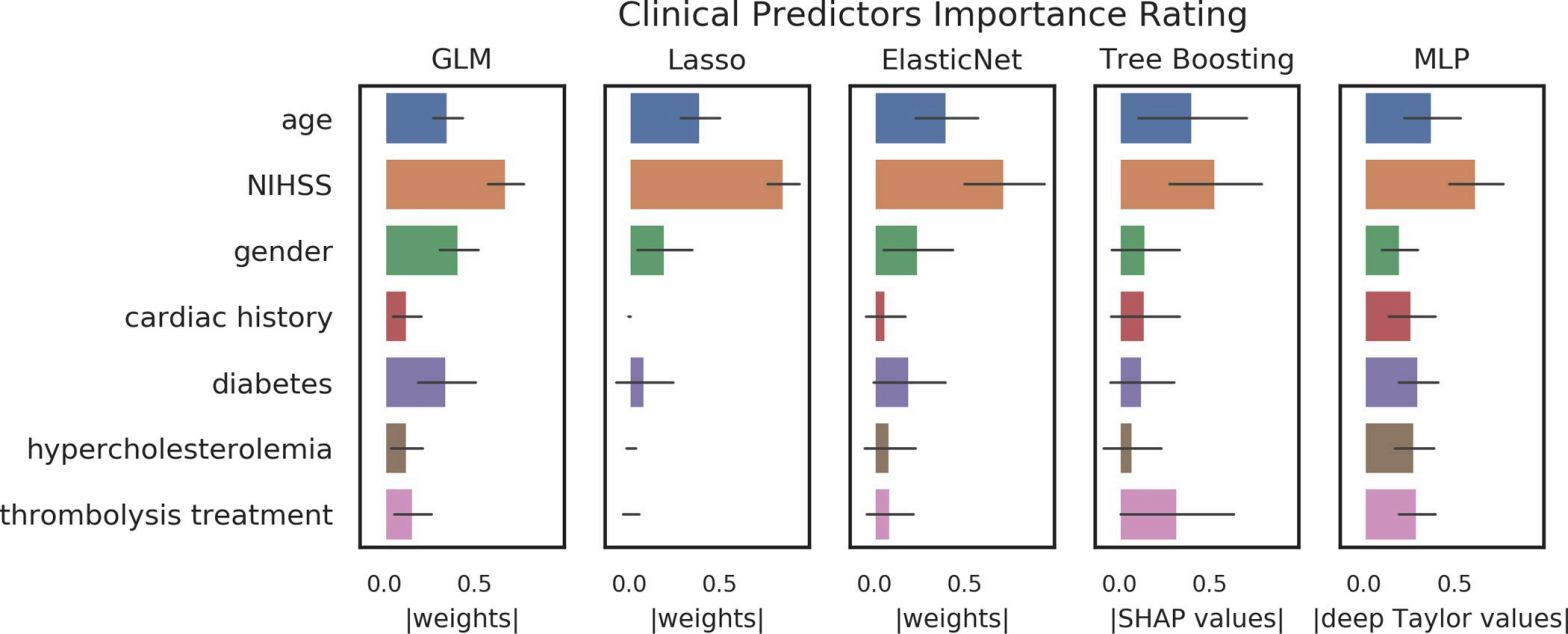
Graphical representation of the feature importance. The figure illustrates the features rating derived from the model-tailored interpretability methods for generalized linear model (GLM), Lasso, Elastic net, Catboost and multilayer perceptron (MLP). All models rated age and initial NIHSS consistently amongst the most important features. For less important features, results were more varied. For logistic regression techniques the results are given in weights, for Catboost in Shap(ley) values and for MLP in deep Taylor values that were normalized to the range [0,1]. The bar heights represent means and error bars represent standard deviation over samples (shuffles).

The interpretability analysis demonstrated consistent results across models in terms of the strongest and established predictors: All explainable models rated age and initial NIHSS consistently amongst the most important features. For less important features, results were more varied. The most similar ratings were obtained between the Elastic net and the tree boosting model. The lowest variance amongst feature importance was found for the MLP model. A graphical representation of the results can be found in Fig 2.

## Discussion

In the present work, we have used a well-characterized clinical stroke outcome prediction paradigm to compare the ability of modern and traditional machine learning methods to provide explainability of their predictions. In the context of the presented study, both types of ML methods (artificial neural nets and tree boosting) showed comparable performance and similar interpretability patterns for the most important predictors. We corroborated that modern techniques are not necessarily black boxes, but are able to provide a reliable assessment of feature importance comparable to their traditional counterparts for clinical prediction models.

In contrast to other domains, models in healthcare require higher levels of safety given that patients’ life and health is at stake [33]. Here, the explainability of the predictions is a highly important criterion to enable it. Unfortunately, explainability in the modeling context is an ill-defined term that can also have other meanings and several other terms such as interpretability and transparency are in use [34]. A comprehensive overview is beyond the scope of the current work, but we would like to introduce two examples. Doran et al. define interpretability as methodological explainability, e.g. the weights of a linear regression algorithm, in contrast to comprehensibility which is a symbolic representation of an output [35]. This view focuses on different users. Interpretability methods can aid developers in the development process, e.g. as a means to find and avoid mistakes. Comprehensible explainability on the other hand refers to how the results are presented to the user in the final product. In healthcare, the users are healthcare professionals with very limited understanding of the technical background of prediction models. Thus, the exact nature of this presentation must be determined—on a case by case basis—for each product. In some cases, more technical presentations as also shown in Fig 2 might suffice. For others, it might be necessary to translate the rankings into easier to understand formats, e.g. categories (“very important” vs. “important” vs “unimportant”). To determine these characteristics is the domain of User experience/User interface (Ux/Ui) analysis, where a thorough testing with users must be performed. This view defines interpretability as a sub-category of explainability. This view defines interpretability as a sub-category of explainability. Others see a distinction. Rudin defines interpretability as an attribute of a method, i.e. a method which inherently provides information about feature importance, such as the weights of linear regression [36]. Explainability on the other hand describes a model which is used to approximate the original model to derive a surrogate interpretability. Such methods can be tailored to one specific original black box algorithm, or can be generalized like the LIME algorithm [37]. We would like to stress that no standardization of these terms currently exists. Thus, in the presented work, explainability is mainly examined from a clinical point-of-view, highlighting the ability of humans to understand which clinical features drive the prediction. This is important, as a major goal of clinical predictive modeling is the development of clinical decision support systems (CDSS) aiding healthcare professionals in their clinical decision making, predicting diagnoses, risks, and outcomes [2,3]. Here, it is important to keep in mind that the requirements for CDSSs go far beyond the model performance [33]. It is established that CDSSs for the clinical setting need to exhibit proven safety [13]. A crucial part of the safety assessment of ML/AI products is to understand why they do what they do, but, more importantly, to understand why and when they might *not* do what is intended. This is important in the light of the increasing awareness of potential biases in models used for healthcare discriminating based on for example sex and gender or ethnicity [38]. Another reason is automation bias—an established cognitive bias—where users tend to believe what a machine is outputting without reflecting on the output [2]. Providing model explainability might mitigate this bias. Thus, it is very likely that future regulatory requirements, e.g. by European MDR and US FDA, will include requests for explainability [39]. Here, our results are highly encouraging. Modern ML methods that are able to provide the potentially highest performance can be combined with methods of explainability and the results are comparable to the established methods for traditional techniques. Thus, researchers and developers are no longer faced with the potential trade-off between lower performance vs. explainability.

However, not only regulatory bodies will require explainability. From the physician point-of-view, black-box approaches might be unacceptable [13,33]. Clinical guidelines for CDSS may therefore profit from explainable predictions. While it has been argued that we have accepted similar uncertainty in medical decision making to date and accuracy alone can be sufficient [40], we would argue that explainability is a must-have when it can be added without limiting the accuracy, as our results suggest. Nonetheless, explainability is a supportive tool and is not a substitute for rigorous clinical validation of any CDSS[40].

We have focused in our work on two promising techniques, namely artificial neural nets and tree boosting. ANNs have shown highly promising results in several areas of healthcare such as medical imaging, information extraction from medical texts and electronic health records, and combining several types of input into one predictive model [5]. Also tree boosting has shown high performance across several medical domains [41]. Tree boosting algorithms are also much easier to train than artificial neural nets and their performance is quite immune to feature scaling and collinearity issues. Another major advantage of tree boosting in healthcare is scalability [42] and thus it is also suited for big data analytics, for example data mining from electronic health records (EHR). Here, tree boosting can achieve comparable performance to deep learning techniques [43]. As evidenced by the above, tree boosting and ANNs represent very versatile and well performing modern ML algorithms in healthcare. Thus, our work is of high practicality for future research and for clinical decision support development.

The main focus of our work was the comparison of explainability in a well-characterized prediction paradigm and not a comparison of performance. It is not surprising that both the traditional and the modern ML methods achieved comparable performance in our dataset. Given the simplicity of the classification problem and the limited dataset, traditional methods are sufficient to capture the relationship of the features to the prediction and complex methods may easily result in overfitting. It is, however, important to note that interpretability without a certain performance level is meaningless: A randomly classifying classifier cannot provide reliable feature importance. If, however, the performance of modern ML methods were considerably higher and the methods´ explainability were to be more reliable, it cannot be determined whether this increase resulted due to a better explainability method or due to a performance increase. Thus, the simplicity of the paradigm we chose is well suited to compare explainability, as the performance is comparable and feature ratings provide a straight-forward result that can be assessed against domain knowledge. Had the performance varied considerably, interpretation of the rankings might have been severely impaired. With regard to our explainability analysis, several more observations are noteworthy. As there is no gold-standard to interpret rankings it can only be performed against domain-knowledge and through replication studies. While we know from previous studies that age, NIHSS and thrombolysis are important predictors to predict stroke outcome (with age and NIHSS being the two strongest) [16–19], it is crucial to include the specifics of the dataset into the interpretation. The median NIHSS of the sample was only 3 and only around 31% of patients received thrombolysis, meaning that many of the patients had smaller—less serious—stroke events. As a consequence, the potential effect of thrombolysis is limited in our sample. Thus we would—like in the above mentioned previous works—expect that age and NIHSS drive the prediction. And indeed, all rankings gave these two very high importance, with the exception of the GLM ranking they were the two most important predictors. The ranking of the lesser predictors, however, varied relatively strongly. Interestingly, elastic net provided the ranking which is most similar to the one provided by tree boosting. From a domain perspective, the most reliable and complete ranking was provided by the tree boosting model, ranking age and NIHSS unequivocally on top, with thrombolysis being slightly more important than the other features. While the MLP gave age and NIHSS the expected high importance, it ranked the presence of diabetes similarly strong. A similar ranking for diabetes can also be observed in the logistic regression models. Although diabetes is known to be an important predictor for bad stroke outcome [44], a feature importance score that is at a similar level as age is unexpected. Another striking difference is the high relative importance given to sex by the logistic regression models, which is absent in the rankings provided by the modern methods. Taken together, we observed promising consistent findings, where all methods corroborated the importance of age and NIHSS for stroke outcome prediction. At the same time, we saw distinct differences for diabetes and sex which cannot be explained sufficiently at the current time point. In light of these findings, we certainly do not claim that the explanations provided by the modern methods should be taken without further validation. Our work established that rankings can be obtained for modern machine learning methods and that these rankings are compatible with clinical interpretation, especially regarding the main predictors. The differences between the rankings, however, must be the subject of further research. Here, it must be mentioned that for ANNs multiple other methods than Taylor decomposition exist, which should also be further tested in the future—a task which was beyond the scope of the current work.

Given the aforementioned trade-off between performance and explainability, a distinction between traditional and modern techniques seems justifiable. It carries with it, however, the risk that modern methods are overhyped and used where traditional techniques might perform best. As our results suggest that also modern techniques provide explainability, we would argue that this distinction is irrelevant. Once all important methods for clinical predictive modeling provide validated feature importance we should simply choose the method which seems best suited for the prediction task at hand. We believe that this will greatly facilitate the development of clinical decision support systems.

Our work has several limitations. First, we used only one dataset. Here, our results are promising, but clearly more analyses are warranted to compare rankings provided by modern ML methods with rankings provided by traditional ML methods. Second, to allow comparison with traditional methods, we used a paradigm that utilizes only clinical values. We encourage future works evaluating explainability provided for other data modalities such as imaging.

## Conclusions

For the first time, we established in an empirical analysis on clinical data that modern machine learning methods can provide explainability which is compatible with domain knowledge interpretation and traditional method rankings. This is highly encouraging for the development of explainable clinical predictive models. Future work should validate the explainability methods, further explore the differences between them, and test different predictive modeling frameworks including multiple modalities.
